# Trend and determinants of anemia change among pregnant and/or lactating women in Ethiopia: A multivariate decomposition analysis

**DOI:** 10.1371/journal.pone.0241975

**Published:** 2020-11-18

**Authors:** Melaku Yalew, Bezawit Adane, Yitayish Damtie, Bereket Kefale, Mastewal Arefaynie, Toyeb Yasin

**Affiliations:** 1 Department of Reproductive and Family Health, School of Public Health, College of Medicine and Health Sciences, Wollo University, Dessie, Ethiopia; 2 Department of Epidemiology and Biostatistics, School of Public Health, College of Medicine and Health Sciences, Wollo University, Dessie, Ethiopia; 3 Department of Health Service Management, School of Public Health, College of Medicine and Health Sciences, Wollo University, Dessie, Ethiopia; Institute of Economic Growth, INDIA

## Abstract

**Background:**

Even though anemia was highly targeted in different global strategies, many pregnant and/or lactating women and children were suffering from it and its complications. Besides this, prior trend analysis has not been conducted among pregnant and/or lactating women in Ethiopia. Therefore, this study aimed to assess the trend and determinants of anemia change among pregnant and/or lactating women in Ethiopia.

**Methods:**

The study utilized two consecutive Ethiopia Demographic and Health Survey (EDHS) datasets. A total of 6,106 and 5,641 pregnant and/or lactating women in 2011 and 2016 survey respectively were included in the analysis. The data were analyzed by using Stata version 14.0. Logit based decomposition analysis was done to identify contributing factors for anemia change and statistical significance was determined by using P-value.

**Results:**

The trend of anemia was increased from 19% in 2011 to 29% in 2016 EDHS. The analysis revealed that, 8% of the overall change in anemia was because of the change in women’s composition. Changes in the composition of pregnant and/or lactating women according to region, economic status and tobacco and/or cigarette use were the major sources of the change. Greater than 90% of the increase in anemia was due to differences in the coefficient. Mostly, the change in behaviors of the Amhara population, those who had a history of terminated pregnancy and use tobacco and/or cigarette were the sources of the change.

**Conclusions:**

Anemia among pregnant and/or lactating women was increased against government interventions over the last half-decade in Ethiopia. Programmatic interventions targeting Somali and Dire Dawa regions are still needed to decrease anemia.

## Introduction

Anemia is a decrease in the concentration of hemoglobin or the reduction in oxygen caring capacity of blood as a result of fewer circulating erythrocytes [1, 2]. It is one of the major public health problems affecting nearly one-third of the global population [1]. Although it can occur at all stages of life, the highest risk of mortality and morbidity is on pregnant, lactating women and under-five children [1, 2]. Globally, thirty-two million four hundred thousand (38.2%) pregnant women were found to anemic [3]. A systematic review conducted in Iran showed that the prevalence of anemia was 17.9% [4]. Its burden became worth in developing countries which was 48.7% in South East Asia and 46.3% in Africa [3]. The prevalence of anemia among pregnant women was 19.7% in Mekelle [5], 14.9% in Adama [6], 10% in Debre Birhan [7] and 32.4% in Dembia, Ethiopia. It was also reached to 24.3% in immediate postpartum or breast feeding women in a study conducted in Debre Markos, Ethiopia [8]. According to the 2016 Ethiopian Demographic and Health Survey (EDHS) data, the prevalence of anemia in reproductive-age women was 24% [9].

It has a major public health consequence on social and economic development resulting in a loss of billions of dollars [10]. For instance, those anemic pregnant women were found more likely to develop antepartum and postpartum hemorrhage and abnormal puerperium especially infection [11, 12]. It may also result in a miscarriage [13] and preterm delivery [14]. Anemia during pregnancy and breastfeeding affects not only mothers but also has an impact on their children. Those children born from anemic women were found more likely to be stunted and underweighted, low birth weight and intrauterine growth restriction [14–18]. It also found to cause perinatal death [12, 19–22], delay in cognitive and motor development [23–25] and dementia [26]. Anemia in the mother during pregnancy also resulted in anemia in the newborn baby [27, 28].

Anemia can be influenced by different factors such as maternal socio-demographic factors (age, educational status, occupation and region) [29–34], husband factors (educational status) [35], behavioral factors (use of tobacco and/or cigarette) [36–39] and obstetric factors (parity, history of abortion) [31, 37, 40–46]. Despite efforts made by the government and other stakeholders, it is still the leading cause of indirect maternal mortality in Ethiopia. Therefore, reducing anemia is one of the opportunities to decrease any pregnancy-related maternal morbidity and mortality that enables to achieve Sustainable Developmental Goals (SDGs) and Growth and Transformation Plan II (GTPII) [47].

Even if anemia was previously studied in different parts of Ethiopia, those studies were only localized to certain settings and their sample size was too small [31, 37, 40–46]. Besides this, prior trend analysis has not been conducted in Ethiopia. So, this study aimed to assess the trend and determinants of anemia change among pregnant and/or lactating women in Ethiopia by using the 2011 and 2016 Demographic and Health Survey data.

## Materials and methods

### Data source and study area

**Fig 1:**
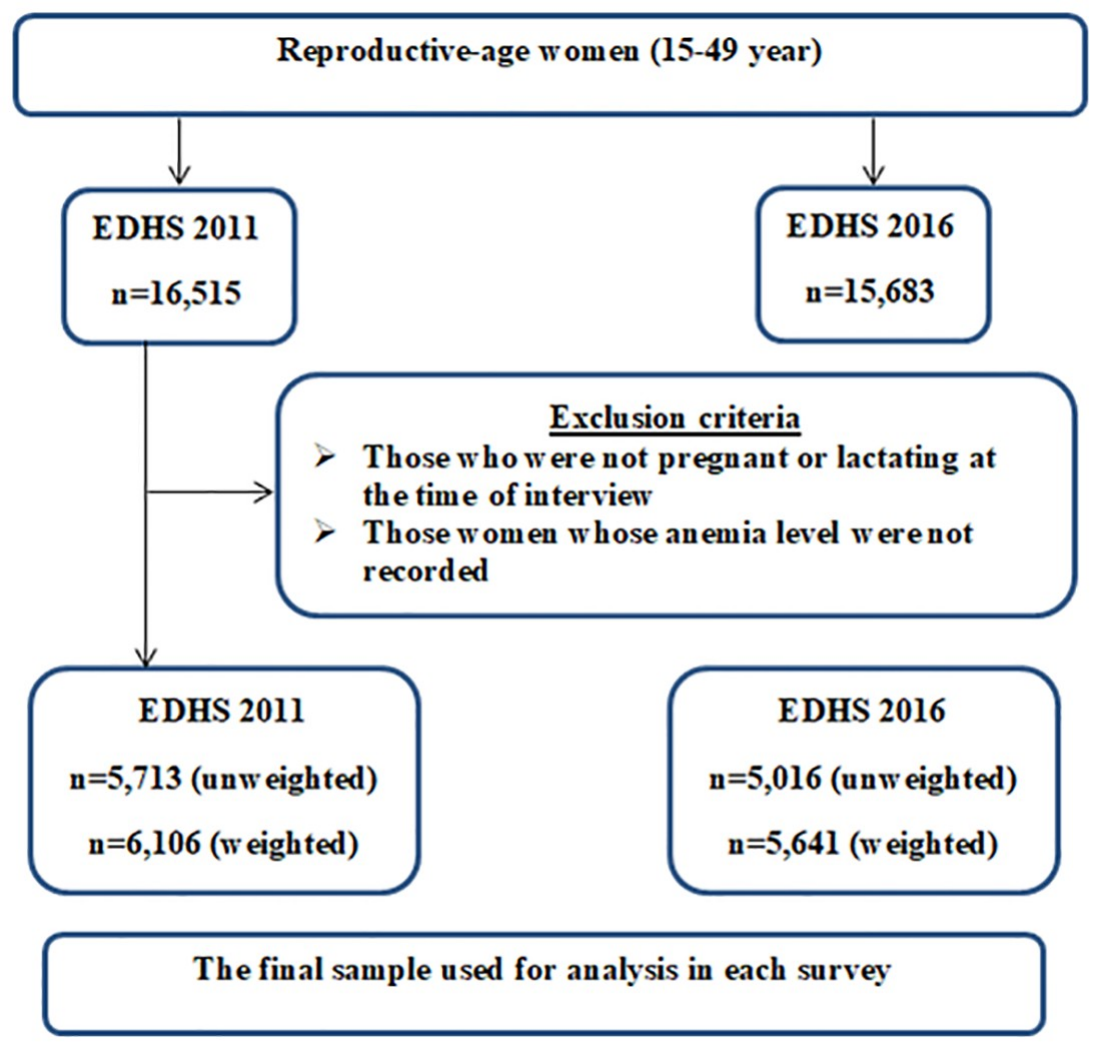
Sampling and exclusion procedures to identify the final sample size in 2011 and 2016 EDHS.

The study was conducted in Ethiopia which is found in the horn of Africa. It utilized datasets of 2011 and 2016 EDHS which were collected by the Central Statistical Agency (CSA) in coordination with the Federal Minister of Health (FMoH) and Ethiopia Public Health Institute (EPHI). The survey used the Ethiopia Population and Housing Census as sampling frame and stratified multistage sampling technique was used. The data were only restricted for pregnant and/or lactating women whose hemoglobin level was recorded. The sample sizes for each survey were 6,106 in 2011 and 5,641in 2016 EDHS (Fig 1).

### Variable measurement

The dependent variable (anemia) was classified dichotomously as “YES/NO” and its classification was different for pregnant and lactating women. Pregnant women whose hemoglobin level less than 11g/dl after adjusted for sea level were categorized as “YES”, otherwise “NO”. Similarly, lactating women whose hemoglobin level less than 12g/dl after adjusted for sea level were categorized as “YES”, otherwise “NO” [9, 48].

### Data quality control and analysis

An initial exploratory data analysis was conducted to check for outliers, missing and consistency. All the results of this study were weighted for sampling probabilities using the weighting factor in the EDHS data. The complex sampling procedure was also considered by using the SVY STATA command to control the clustering effect of complex sampling. The data were analyzed by using Stata version 14.0 after the two datasets were merged by using the Stata command “append”. Multivariate decomposition analysis was done to see the change in anemia and its contributing factors for the change. The purpose of decomposition analysis was to identify the source of change in anemia among pregnant and lactating women in the last half-decade (2011 to 2016). The analysis used the output for the logistic regression model to decompose the observed difference in anemia into two components. The first change was due to variation in population structure or composition across the survey. The second change was due to change in the behavior of the survey population as the change in outcome was due to either change in population composition or change in behavior of the population or both. The observed differences in anemia levels between different surveys were decomposed into characteristics (natural endowment or population composition) and a coefficient (effect of characteristics or behavioral effect). The logit based difference can be decomposed as [49].

$$Y=F(\frac{e^{X\beta }}{1+e^{X\beta }})+\varepsilon$$ (1)

$$Ya-Yb=F(\frac{e^{Xa\beta a}}{1+e^{Xa\beta a}})-F(\frac{e^{Xb\beta b}}{1+e^{Xb\beta b}})+\varepsilon$$ (2)

$$\Delta Y=[F(\frac{e^{Xa\beta a}}{1+e^{Xa\beta a}})-F(\frac{e^{Xb\beta a}}{1+e^{Xb\beta a}})]+[F(\frac{e^{Xb\beta a}}{1+e^{Xb\beta a}})-F(\frac{e^{Xb\beta b}}{1+e^{Xb\beta b}})]+\;\varepsilon$$ (3)

Where: Y is the dependent variable, X is the independent variable, *β* is the coefficient and F is differential logistic function of $X(\frac{e^{X\beta }}{1+e^{X\beta }})and\;Y.$ Hence, the result focused on how anemia responded to population composition and their behavior and how these factors shape it across different surveys at different times. The level of statistical significance was set at a P value of less than 0.05.

### Ethical considerations

The data were accessed from CSA by requesting it through a web site www.measuredhs.com. Then, authorization letter was received from CSA to download EDHS dataset. The data were used only for this study and it was not passed to other researchers. All data were treated as confidential and no personal or household identifiers were used in the survey. The detailed information on ethical issues was published within the EDHS report [9].

## Results

### Characteristics of the respondents

Table 1 represents the characteristics of study participants over the two consecutive EDHS. Among the total respondents, more than twenty percent of them were in ages between 20–24 years in both surveys. Whereas, the proportion of respondents whose ages between 30–34 years were increased from 18% in 2011 to 22% in 2016 EDHS. Concerning the educational status of women, about 4% and 8% of pregnant and/or lactating women were found to be educated secondary school and above in 2011 and 2016 respectively. At the same time, the educational status of their husbands was also increased from 48.85% to 53.5% from 2011 to 2016. However, the proportions of orthodox followers were decreased from 2011 to 2016 (41% to 38%). Concerning the occupational status of women, about 47% in 2011 and 55% in 2016 were found to be not working at the time of the survey. The proportions of women who belong in all categories of wealth quintiles were almost constant for both surveys. The mean total numbers of children ever born were almost similar across both surveys which were 4. Greater than 90% of the respondents didn’t have a history of terminated pregnancy in both surveys. At the time of the interview, one out of the five participants was pregnant in both surveys (Table 1).

**Table 1:** Percent distribution of characteristics of participants, in 2011 & 2016 Ethiopia demographic and health surveys.

Characteristics	Category	EDHS 2011, n = 6,106% (95% CI)	EDHS 2016, n = 5,641% (95% CI)
**Age of women in years**	15–19	7.10 (6.48, 7.77)	6.09 (5.49, 6.74)
	20–24	21.72 (20.70, 22.77)	22.33 (21.26, 23.44)
	25–29	30.91 (29.77, 32.08)	28.31 (27.15, 29.50)
	30–34	18.16 (17.21, 19.14)	22.16 (21.10, 23.27)
	35–39	14.35 (13.50, 15.26)	13.82 (12.95, 14.75)
	40–44	6.15 (5.58, 6.78)	5.64 (5.07, 6.28)
	45–49	1.60 (1.31, 1.95)	1.64 (1.34, 2.01)
**Marital status**	Single or not in union	6.67 (6.07, 7.33)	4.58 (4.06, 5.16)
	Married or living together	93.33 (92.67, 93.93)	95.42 (94.84, 95.93)
**Place of residence**	Urban	11.94 (11.15, 12.77)	11.30 (10.50, 12.16)
	Rural	88.06 (87.22, 88.85)	88.70 (87.84, 89.50)
**Educational status of women**	Not educated	67.69 (66.50, 68.85)	60.78 (59.50, 62.04)
	Primary	28.43 (27.31, 29.57)	30.96 (29.77, 32.18)
	Secondary	2.58 (2.21, 3.00)	5.96 (5.38, 6.61)
	College and above	1.31 (1.05, 1.63)	2.30 (1.93, 2.72)
**Educational status of husband**	Not educated	51.15 (49.89, 52.40)	46.50 (45.17, 47.83)
	Primary	40.96 (39.73, 42.21)	40.22 (38.92, 41.54)
	Secondary	4.89 (4.37, 5.46)	8.76 (8.03, 9.54)
	College and above	3.00 (2.60, 3.46)	4.53 (4.00, 5.12)
**Occupation status of women**	Not working	46.81 (45.56, 48.06)	54.85 (53.55, 56.14)
	Working	53.19 (51.93, 54.44)	45.15 (43.86, 46.45)
**Religion**	Orthodox	40.65 (39.42, 41.89)	37.93 (36.67, 39.21)
	Protestant	23.73 (22.67, 24.81)	21.80 (20.74, 22.90)
	Muslim	32.49 (31.32, 33.67)	37.11 (35.85, 38.37)
	Others	3.14 (2.73, 3.61)	3.16 (2.74, 3.65)
**Region**	Tigray	5.86 (5.30, 6.48)	6.64 (6.02, 7.32)
	Afar	0.82 (0.62, 1.08)	0.80 (0.60, 1.07)
	Amhara	25.07 (24.00, 26.17)	22.99 (21.91, 24.10)
	Oromia	40.54 (39.31, 41.77)	40.80 (39.53, 42.09)
	Somali	2.12 (1.79, 2.52)	3.12 (2.69, 3.60)
	Benishangul Gumuz	1.21 (0.96, 1.51)	1.07 (0.83, 1.37)
	SNNP	21.69 (20.68, 22.75)	21.67 (20.61, 22.77)
	Gambela	0.36 (0.23, 0.54)	0.24 (0.14, 0.41)
	Harari	0.22 (0.13, 0.37)	0.20 (0.11, 0.35)
	Addis Ababa	1.82 (1.51, 2.18)	2.11 (1.76, 2.52)
	Dire Dawa	0.29 (0.18, 0.46)	0.37 (0.24, 0.57)
**Wealth index**	Poorest	23.37 (22.32, 24.44)	22.52 (21.45, 23.63)
	Poorer	22.37 (21.34, 23.43)	22.62 (21.55, 23.73)
	Middle	21.15 (20.14, 22.19)	21.34 (20.29, 22.43)
	Richer	18.93 (17.97, 19.94)	18.67 (17.67, 19.70)
	Richest	14.19 (13.33, 15.09)	14.86 (13.95, 15.81)
**Number of children ever born**	0	3.21 (2.80, 3.68)	3.78 (3.31, 4.30)
	1–5	70.36 (69.20, 71.49)	71.05 (69.85, 72.22)
	≥ 6	26.43 (25.34, 27.55)	25.17 (24.06, 26.32)
**History of abortion**	No	89.70 (88.90, 90.43)	91.48 (90.73, 92.18)
	Yes	10.30 (9.56, 11.09)	8.52 (7.82, 9.27)
**Current status of women**	Pregnant	19.21 (18.24, 20.21)	19.28 (18.27, 20.33)
	Breastfeeding	80.79 (79.79, 81.76)	80.72 (79.67, 81.73)
**Use of tobacco and/or cigarette**	No	99.23 (98.97, 99.42)	99.25 (98.99, 99.44)
	Yes	0.77 (0.58, 1.03)	0.75 (0.55, 1.01)

Others = Catholic and traditional followers, CI = Confidence Interval.

### Trends of anemia

**Fig 2:**
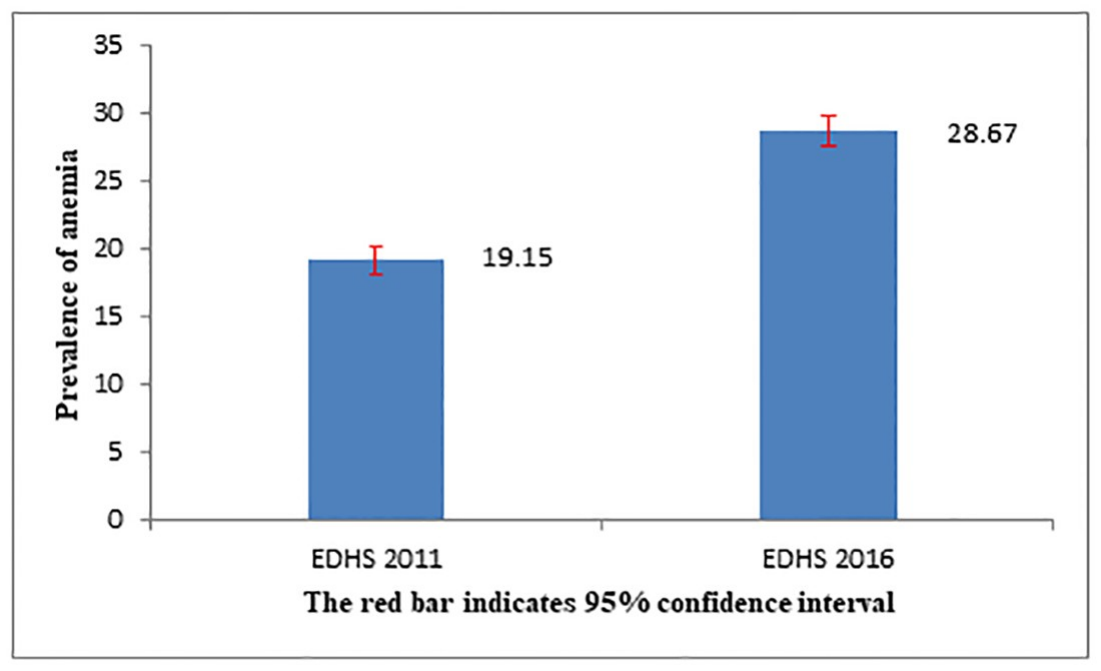
The trends of anemia among pregnant and/or lactating women in five years, EDHS, 2011–2016.

The overall trend of anemia among pregnant and/or lactating women was significantly increased, from 19.15% in 2011 to 28.67% in 2016 (Fig 2).

The trend of anemia among pregnant and/or lactating women showed variation per different respondents’ characteristics. Anemia was increased in pregnant and/or lactating women in all age groups. The highest increment was 13% and the lowest was 3% in the age of 30–34 and 40–44 years respectively. The trend of anemia was increased by 10% in rural dwellers of pregnant and/or lactating women as compared to urban. Similarly, anemia was increased in study participants who were not educated (20% in 2011 to 32% in 2016). In four regions: Somali, Southern Nation Nationalities and Peoples (SNNP), Oromia and Tigray, the level of anemia showed an increment above 10%. Anemia was also increased by 20% in pregnant and lactating women who belong to the poorest wealth quintile (Table 2).

**Table 2:** The trend of anemia among pregnant and/or lactating women by selected characteristics of respondents, 2011 and 2016 EDHS.

Characteristics	EDHS 2011 n = 6,106	EDHS 2016 n = 5,641	Percentage point difference in anemia (2016–2011)
**Age of women in years**			
**15–19**	20.22	26.07	5.85
**20–24**	17.83	27.57	9.74
**25–29**	19.39	28.40	9.01
**30–34**	18.20	31.05	12.85
**35–39**	21.27	30.73	9.46
**40–44**	19.71	22.80	3.09
**45–49**	17.36	28.81	11.45
**Marital status**			
**Single or not in union**	18.89	24.01	5.12
**Married or living together**	19.17	28.90	9.73
**Place of residence**			
**Urban**	13.93	18.41	4.48
**Rural**	19.86	29.98	10.12
**Educational status of women**			
**Not educated**	20.22	31.65	11.43
**Primary**	17.83	25.28	7.45
**Secondary**	10.11	20.79	10.68
**College and above**	10.29	16.03	5.74
**Educational status of husband**			
**Not educated**	21.47	30.99	9.52
**Primary**	17.24	28.34	11.10
**Secondary**	14.30	22.57	8.27
**College and above**	14.61	24.63	10.02
**Occupation status of women**			
**Not working**	21.76	31.02	9.26
**Working**	16.85	25.83	8.98
**Religion**			
**Orthodox**	15.97	22.18	6.21
**Protestant**	14.54	26.70	12.16
**Muslim**	27.20	35.52	8.32
**Others**	11.84	39.76	27.92
**Region**			
**Tigray**	14.61	24.94	10.33
**Afar**	41.39	50.26	8.87
**Amhara**	19.97	21.52	1.55
**Oromia**	20.64	32.49	11.85
**Somali**	49.38	67.60	18.22
**Benishangul Gumuz**	21.95	25.03	3.08
**SNNP**	13.06	24.77	11.71
**Gambela**	25.86	30.91	5.05
**Harari**	25.70	35.67	9.97
**Addis Ababa**	9.26	17.70	8.44
**Dire Dawa**	40.12	40.68	0.56
**Wealth index**			
**Poorest**	21.19	41.22	20.03
**Poorer**	21.42	29.12	7.70
**Middle**	17.90	25.58	7.68
**Richer**	18.51	24.67	6.16
**Richest**	13.74	18.44	4.70
**History of a terminated pregnancy**			
**No**	18.58	29.09	10.51
**Yes**	24.13	24.20	0.07
**Total number of children ever born**			
**0**	16.46	21.92	5.46
**1–5**	18.77	27.67	8.90
**≥ 6**	20.48	32.53	12.05
**Current status of women**			
**Pregnant**	22.03	29.13	7.10
**Breastfeeding**	18.46	28.56	10.10
**Use of tobacco and/or cigarette**			
**No**	19.14	28.78	9.64
**Yes**	20.77	14.74	-6.03
**Total**	19.15	28.67	9.52

Others = Catholic and traditional followers.

### Decomposition analysis

The decomposition analysis model has been taken into account the differences in the characteristics (compositional factors) and the differences due to the effect of characteristics. Only 8% of the overall anemia change was due to the differences in characteristics. Among the compositional factors, a very significant contribution to change in anemia in pregnant and/or lactating women was due to the region in which they lived. The increased in the compositions of women who lived in Somali and Dire Dawa showed a significant contribution to increase in anemia. However, a decreased in the proportion of women who lived in Afar and Harari showed a significant negative impact on anemia. Although compositional changes were too small, an increased in the composition of women who resided in poorer and richer households showed a statistically significant positive impact on anemia change. Another compositional factor affecting anemia change was the use of tobacco and/or cigarette. An increased in the proportion of tobacco and/or cigarette use showed a significant contribution to the change in anemia (Table 3).

**Table 3:** Decomposition of change in anemia among pregnant and/or lactating women in Ethiopia, 2011 to 2016.

Category	Difference due to characteristics (E)			Difference due to coefficients (C)		
	Coefficient	Percent	P-value	Coefficient	Percent	P-value
**Age of women in years**						
**15–19**	^®^					
**20–24**	0.00011	0.110	0.700	0.009	9.276	0.437
**25–29**	-0.00072	-0.740	0.544	0.0099	10.168	0.548
**30–34**	0.002543	2.614	0.311	0.011	11.243	0.297
**35–39**	-0.00032	-0.327	0.440	0.0031	3.139	0.736
**40–44**	0.00020	0.180	0.638	-0.0014	-1.4282	0.769
**45–49**	0.000015	0.015	0.762	0.00055	0.561	0.755
**Place of residence**						
**Urban**	^®^					
**Rural**	0.00021	0.215	0.554	0.00032	0.3256	0.995
**Educational status of women**						
**Not educated**	^®^					
**Primary**	-0.0008	-0.828	0.288	-0.00794	-8.16	0.355
**Secondary**	-0.0014	-1.476	0.528	0.00083	0.856	0.700
**Higher and +**	-0.0015	-1.522	0.177	-0.00062	-0.641	0.682
**Educational status of husband**						
**Not educated**	^®^					
**Primary**	-0.00013	-0.129	0.555	0.0010	10.214	0.386
**Secondary**	0.00046	0.472	0.822	0.0013	1.3614	0.643
**Higher and +**	0.0021	2.13	0.056	0.0021	2.1102	0.393
**Occupation status of women**						
**Not working**	^®^					
**Working**	0.0017	1.7795	0.418	0.0111	11.366	0.398
**Religion**						
**Orthodox**	^®^					
**Protestant**	-0.001	-1.069	0.201	0.0019	1.98	0.849
**Muslim**	0.0027	2.765	0.106	-0.021	-22.01	0.057
**Others**	0.0002	0.169	0.079	0.0036	3.66	0.135
**Region**						
**Tigray**	^®^					
**Afar**	-0.00001	-0.0122	0.014	-0.0002	-0.152	0.737
**Amhara**	0.00122	1.2488	0.163	-0.0213	-21.89	0.029
**Oromia**	-0.000047	-0.0481	0.586	0.0077	7.96	0.662
**Somali**	0.00331	3.407	0.000001	0.00085	0.875	0.461
**Benishangul Gumz**	0.00009	0.095	0.168	-0.00074	-0.758	0.215
**SNNP**	-0.00028	-0.287	0.214	0.00380	3.897	0.721
**Gambela**	-0.00004	-0.045	0.573	-0.00024	-0.252	0.248
**Harari**	-0.00003	-0.029	0.038	0.00008	0.088	0.480
**Addis Ababa**	0.00012	0.119	0.550	0.00170	1.743	0.217
**Dire Dawa**	0.00013	0.129	0.014	-0.00015	-0.151	0.367
**Wealth index**						
**Poorest**	^®^					
**Poorer**	0.00016	0.169	0.079	-0.0159	-16.367	0.037
**Middle**	-0.00010	-0.104	0.003	-0.0132	-13.545	0.083
**Richer**	0.00023	0.232	0.001	-0.0150	-15.436	0.038
**Richest**	-0.00210	-2.146	0.001	-0.0144	-14.814	0.080
**Current status of women**						
**Pregnant**	^®^					
**Breastfeeding**	0.00002	0.0215	0.675	0.0251	25.76	0.325
**History of a terminated pregnancy**						
**No**	^®^					
**Yes**	0.00102	1.045	0.178	-0.0088	-9.01	0.032
**Use of tobacco and/or cigarette**						
**No**	^®^					
**Yes**	0.00042	0.432	0.000001	-0.0032	-3.292	0.00001
**Total number of children ever born**						
**0**	^®^					
**1–5**	0.000084	0.0863	0.705	-0.01397	-14.361	0.784
**≥6**	-0.00029	-0.2976	0.566	0.00019	0.194	0.993
**Total**	.0074	7.5836	0.021	0.0899	92.416	0.000001

Others = Catholic and traditional followers, SNNP = Southern Nation Nationalities and Peoples and ^®^ = Reference.

After controlling the effect of compositional factors, 92% of the change in anemia level was due to the difference in the effects of characteristics. Factors including wealth of the household, history of a terminated pregnancy, tobacco or cigarette use and region in which they lived showed a significant contribution to the observed negative change in anemia. Keeping other things constant, more than one-fifth of the change in anemia was due to the change in behavior among the Amhara population. Likewise, the behaviors of the population who lived in poorer and richer households were contributed to the decreased anemia change. As compared to the behaviors of women who hadn’t a history of abortion, change in the behavior of participants who had a history has shown a significant contribution to the negative anemia change. Similarly, a decreased level of anemia was due to the effect of behavioral changes in those women who used tobacco and/or cigarette (Table 3).

## Discussion

In this study, the trend of anemia was significantly increased over the last half-decade (2011 to 2016) among pregnant and/or lactating women in Ethiopia. The major contributing factors for this change were due to both differences in population composition and changes in the behavior of the population. This finding was higher than the trend of anemia among all reproductive-age women in Ethiopia [9]. But, it was lower than studies conducted in Ghana, low and middle-income countries (LMICs) and India [50–53]. Still, it was lower than the global prevalence of anemia among pregnant and non-pregnant women [54]. But, it was higher than the trends of anemia among women aged 20–49 years in United State [55]. This might be due to poor response and cooperation of the population for any anemia prevention strategies which has been practiced before (distributing insecticide-treated net and parasite disinfestation or mass deworming). Moreover, it might be due to poor practice of preconception care in Ethiopia [56]. Even the prevalence of good adherence to iron/folic acid supplementation during pregnancy and immediate postpartum was low which may contributed to increase anemia among pregnant and/or lactating women [57, 58].

Even though the contribution to anemia change was small, changes in population composition were one of the determinant variables in a positive and negative direction. About one in twelve changes in anemia was due to differences in the characteristics. This implied that a significant contribution to anemia change was due to the change in population composition across surveys.

Among the compositional factors, a very significant contribution to the positive change of anemia was due to the Somali region. The increased women composition that lived in Somali and Dire Dawa showed a significant contribution to positive change (increase) in anemia. This finding was consistent with a study conducted in Ghana [50]. An increase in the compositions of women who resides in poorer households showed a statistically significant contribution to positive anemia change. This finding was similar to a study conducted in India [53]. It reflected that the more they are economically developed, the less risk of being anemic which should be emphasized to the future. Another compositional factor contributed to anemia change was the use of tobacco and/or cigarette. An increase in the proportion of use of tobacco and/or cigarette showed a significant contribution to anemia change in pregnant and/or lactating women. This may be due to the natural feature of the substance present in tobacco and/or cigarette to attract oxygen than hemoglobin.

After controlling the effect of compositional factors, nine out of ten changes in anemia level were due to the difference in the effects of characteristics. More than one-fifth of change in anemia in the past five years was due to the change in behavior among the Amhara population. Likewise, changes in the population behavior that belong to richer households contribute to a decreased level of anemia. As compared to the behaviors of women who hadn’t a history of abortion, change in the behavior of participants who had a history has shown a significant contribution to the negative anemia change. Again, a change in the behavior of women who used tobacco and /or cigarette significantly associated with a negative change in anemia. Even if the study utilized large datasets and considers sampling weighing, it is not without limitation. As the two surveys were not conducted in the same participants, in was not real time series analysis. The decomposition analysis only considered those pregnant and/or lactating women whose anemia was recorded. In addition, the analysis included only variables recorded in both surveys and those included factors are not the only factors that could affect anemia.

## Conclusions

The trend of anemia was significantly increased among pregnant and/or lactating women over the last half-decade in Ethiopia. The decomposition analysis indicated that the overall change in anemia was due to the change in women’s composition and behavior related to the population. Change in the composition of pregnant and/or lactating women’s characteristics according to region, economic status and tobacco or cigarette use were the major sources of the change. The majority of the increase in anemia was due to differences in the coefficient. Mostly, the change of behaviors on the Amhara population, those who had a history of terminated pregnancy and use of tobacco and/or cigarette were the sources of the change. Programmatic interventions targeting richer households, Somali and Dire Dawa regions are still needed to decrease anemia.

## Abbreviations

CSA: Central Statistical Agency
EDHS: Ethiopia Demographic and Health Survey
WHO: World Health Organization
